# Venous thromboembolic risk in hematological hospitalized patients: a retrospective study

**DOI:** 10.1007/s00277-025-06397-9

**Published:** 2025-05-06

**Authors:** Stefano Cordella, Valeria Coluccio, Riccardo Cuoghi Costantini, Roberto D’Amico, Mario Luppi, Marco Marietta

**Affiliations:** 1 Hematology Unit, Azienda Ospedaliero-Universitaria, 41124 Modena, Italy; 2 Department of Medical and Surgical Sciences, Section of Hematology, University of Modena and Reggio Emilia, 41124 Modena, Italy; 3 Clinical and Experimental Medicine PhD Programme, Department of Biomedical, Metabolic and Neural Sciences, University of Modena and Reggio Emilia, Modena, 41125 Italy; 4 Unit of Statistical and Methodological Support to Clinical Research, Azienda Ospedaliero-Universitaria, 41124 Modena, Italy; 5 Department of Medical and Surgical Sciences for Children and Adults, University of Modena and Reggio Emilia, 41124 Modena, Italy; 6 Department of Oncology and Hematology, Azienda Ospedaliero-Universitaria, 41124 Modena, Italy

**Keywords:** Venous thromboembolism, Prophylaxis, Major bleeding, Hematological diseases, PADUA score

## Abstract

Although patients with hematological malignancies carry a non-negligible risk of venous thromboembolic events (VTE), no risk assessment models (RAMs) have been developed and validated to predict this risk during their hospital stay. Moreover, the performance of RAMs developed to predict the thromboembolic and hemorrhagic risk in hospitalized medical patients has never been assessed in this specific population. In this observational study we sought to assess the incidence of VTE and hemorrhagic events (HE), to evaluate pharmacological prophylaxis use, and to test existing RAMs for TE and HE (Padua Predictions Score, PPS, and Improve Bleeding Score, IBS, respectively) in a population of 514 hematological patients at their first hospital admission. 165 patients were found to be at high thromboembolic and 114 at high hemorrhagic risk according to PPS and IBS; a total of 148 patients received pharmacological prophylaxis with low-molecular weight heparin (LMWH). An incidence of VTE (3.11%) and HE (1.75%) was found, with most thrombotic (15/16) and hemorrhagic (8/9) events occurring in patients not receiving prophylaxis. At the univariate analysis, the diagnosis of acute leukemia was significantly associated with an increased risk of VTE, whereas prophylaxis was found to be protective. However, at the multivariate analysis the statistical significance was lost for both of these variables (HR = 2.76, *p* = 0.218 and HR = 0.22, *p* = 0.170, respectively). PPS poorly performed in this population, but no other factors associated with VTE were found. Larger, multicenter studies to develop better RAMs tailored to HM patients are needed to improve VTE management in this population.

**Supplementary Information:**

The online version contains supplementary material available at 10.1007/s00277-025-06397-9.

## Introduction

Venous thromboembolism (VTE) is a common and serious complication in cancer patients, first described by Armand Trousseau in the 1860s [1–4]. The strong association between cancer and thrombosis also applies to hematological malignancies (HM): it is estimated that up to 10% of patients with HM, excluding those with myeloproliferative neoplasms, may experience a VTE event within 6 to 12 months of diagnosis [5–7]. The prevalence of thrombotic events is even higher in patients with myeloproliferative neoplasms, and it has been demonstrated that the development of VTE negatively affects the prognosis of patients with HM [5, 8–12]. Moreover, also non-malignant haematological disease, such as Immune thrombocytopenia and thrombotic thrombocytopenic purpura, carry a relevant risk of VTE [13, 14].

Several risk assessment models (RAMs) have been developed and validated to stratify VTE risk in hospitalized or ambulatory cancer patients, particularly those with solid tumors, with moderate to good predictive ability [15–21]. However, fewer than 10% of patients included in these models had HM, limiting their reliability in this specific population. Some RAMs specifically for patients with HM have been developed, though only one focused on the hospital stay [22], while others reported overall VTE risk during longer follow-up periods [23–30].

On the other hand, RAMs for estimating thrombotic and hemorrhagic risk in hospitalized non-surgical patients have been developed and validated [31]. Among them, the Padua Prediction Score (PPS) [32] for thrombotic risk and the Improved Bleeding Score (IBS) [33] for hemorrhagic risk are the most widely used in clinical practice. However, their performance in patients with hematological diseases (HD) has never been assessed.

On these premises, a reappraisal of the VTE issue in hospitalized patients HD, either malignant or benign, is advisable to better estimate the VTE incidence in this specific setting during hospital stay, to identify thrombosis and bleeding risk factors, and to assess the use of pharmacological prophylaxis in clinical practice.

Therefore, we conducted a retrospective study with the following aims: (i) to assess the incidence of VTE and major bleeding; (ii) to evaluate pharmacological prophylaxis use; (iii) to assess PPS and IBS performance for predicting VTE and major bleeding; and (iv) to identify factors associated with VTE and major bleeding in hospitalized HD patients.

## Methods

We retrospectively identified consecutive patients who were admitted for the first time to our hematology division between January 1, 2016, and December 31, 2022, with a confirmed diagnosis of HD.

Only hospitalizations lasting at least 2 days were considered. At admission, in addition to standard demographic factors and routine tests, data related to ongoing therapies, as well as the PPS and IBS values, were collected through the electronic medical records used in our department.

The exclusion criteria were as follows: (i) Ongoing anticoagulant therapy at dosages higher than standard prophylaxis (as defined below) for more than 50% of the hospitalization period; (ii) Lack of thromboembolic and bleeding risk assessments; (iii) In the previous month, occurrence of a thromboembolic event, either arterial or venous, or clinically relevant bleeding.

The decision to use pharmacological prophylaxis was made by the attending physician for each patient. Standard prophylaxis consisted of enoxaparin (LMWH) at a dose of 4000 IU/day, or 6000 IU/day for patients with a Body Mass Index (BMI) greater than 30. In patients with renal dysfunction (defined as a creatinine clearance below 30 mL/min), either 2000 IU/day of LMWH or unfractionated heparin (UFH) at a dose of 5000 IU, administered two or three times a day, was used. The dose of LMWH/UFH was reduced by 50% if the platelet count (PLTc) was between 25 and 50 × 10^9/L, and was discontinued or not initiated if the PLTc was below 25 × 10^9/L.

VTE was defined as any instrumentally proven venous thrombosis, including incidentally detected pulmonary embolism and proximal or distal deep vein thrombosis of the legs. Deep vein thrombosis related to central venous catheters was included in the analysis.

Hemorrhagic event (HE) was defined as any bleeding event requiring a blood transfusion of at least one unit of Red Blood Cells within 2 h of the event (a surrogate for a Grade 3 event in the Common Terminology Criteria for Adverse Events classification), or any bleeding involving the central nervous system.

### Statistical analysis

Continuous variables were reported as median and interquartile range (IQR) or mean and standard deviation (SD), while categorical variables were reported as absolute and percentage frequencies. The associations between Prophylaxis and patients’ characteristics were assessed using Student t test or Wilcoxon-Mann-Whitney test for continuous numerical variables, whereas Pearson’s Chi square test or Fisher Exact test were used for categorical variables. Statistical comparisons were not performed for covariates in which one of the groups had no observed events, in order to avoid misleading interpretations.

Probability of receiving thromboprophylaxis according to PPS and IBS was shown in Heatmap.

Survival analysis was carried out to investigate the relationship between occurrence of TE and other variables. A comprehensive univariable analysis, considering demographic characteristics as well as the items included in the PPS, was initially performed using Cox Proportional Hazard models. Subsequently, a multivariable model was estimated, considering the variables found to be statistically significant in the univariable analysis.

The results were reported as Hazard Ratios (HRs) with 95% Confidence Intervals (95% CIs) and p-values. The performance of the PPS in predicting the occurrence of VTE was assessed using the time-dependent area under the ROC curve. P-values were considered statistically significant when below the alpha level set at 0.05.

All analyses were carried out using R version 4.3.2 (The R Foundation for Statistical Computing, 2023).

## Results

A total of 514 patients (282 males, 232 females) with a new diagnosis of HD were admitted for the first time to our division during the study period. The baseline demographics, disease characteristics, and PPS and IBS values of the study participants are detailed in Table 1. Acute leukemia was the most common diagnosis (226 patients, 44% of cases), followed by Lymphoma (173 patients, 33% of cases) and Multiple Myeloma (67 patients, 13% of cases).

**Table 1:** Baseline patients characteristics

	Overall *N* = 514	No Prophylaxis *N* = 362	Prophylaxis *N* = 152	*p*
Sex M/F	282/232	200/162	82/70	0.787
Age (years), median (IQR)	58 [47-66.75]	56 [46–66]	59 [49.75-67]	0.099
PLT at admission (x10^3), median (IQR)	153 [47–253]	118 [38–227]	202 [109.50-295.25]	*< 0.001*
Hospital stay (days), median (IQR)	34 [10–39]	26.5 [10–38]	22 [10–42]	0.534
**Hematological diseases**				
AML (except APL), n. (%)	158 (31)	142 (39)	16 (11)	*< 0.001*
APL, n. (%)	20 (4)	20 (6)	0	*-*
ALL, n. (%)	48 (9)	42 (11)	6 (4)	*0.006*
Lymphoma, n. (%)	173 (33)	86 (24)	87 (57)	*< 0.001*
Multiple Myeloma, n. (%)	67 (13)	35 (10)	32 (21)	*< 0.001*
Other:				
− malignant (ex. MPNs), n. (%)	24 (5)	16 (4)	8 (5)	0.653
− non-malignant (ex. autoimmune cytopenias), n. (%)	24 (5)	21 (6)	3 (2)	0.068
**Padua Prediction Score (PPS)**				
PPS, median (IQR)	3 [3–4]	3 [3–3]	4 [3–6]	*< 0.001*
PPS ≥ 4, n. (%)	165 (32)	78 (22)	87 (57)	*< 0.001*
Items contributing to PPS:				
Heart and/or respiratory failure, n. (%)	15 (3)	8 (2)	7 (5)	0.141
Acute myocardial infarction, n. (%)	8 (2)	5 (1)	3 (2)	0.699
Ischemic stroke, n. (%)	1 (0)	1 (0)	0	-
Acute infection, n. (%)	68 (13)	42 (12)	26 (17)	0.093
Obesity, n. (%)	50 (10)	28 (8)	22 (15)	*0.019*
Hormonal treatment, n. (%)	6 (1)	6 (2)	0	-
Recent trauma/major surgery < 1 month, n. (%)	10 (2)	1 (0)	9 (6)	*< 0.001*
Active neoplasm, n. (%)	490 (95)	341 (94)	149 (98)	0.068
Previous VTE, n. (%)	16 (3)	4 (1)	12 (8)	*< 0.001*
Reduced mobility (bedrest > 2 days), n. (%)	41 (8)	4 (1)	37 (24)	*< 0.001*
Thrombophilia (congenital or acquired), n. (%)	10 (2)	7 (2)	3 (2)	1
**Improve Bleeding Score (IBS)**				
IBS, median (IQR)	4.5 [3.5–6.5]	4.5 [3.5-7]	4.5 [3.5-5]	*0.001*
IBS ≥ 7, n. (%)	114 (22)	97 (27)	17 (12)	*< 0.001*
Items contributing to IBS:				
eGFR 30–59 ml/min, n. (%)	21 (4)	18 (5)	3 (2)	0.146
eGFR < 30 ml/min, n. (%)	13 (3)	4 (1)	9 (6)	*0.003*
Rheumatic disease, n. (%)	6 (1)	6 (2)	0	-
Central venous catheter, n. (%)	60 (12)	40 (11)	20 (13)	0.497
ICU admission, n. (%)	4 (1)	1 (0)	3 (2)	0.080
Liver failure, n. (%)	3 (1)	2 (1)	1 (1)	1
PLT < 50 × 10^3, n. (%)	126 (25)	113 (31)	13 (9)	*< 0.001*
Major bleeding < 3 months, n. (%)	4 (1)	3 (1)	1 (1)	1
Active gastroduodenal ulcer, n. (%)	5 (1)	5 (1)	0	-

Patients with high thrombotic and haemorrhagic risk were defined as those having a PPS > 4 and an IBS > 7, as previously described [29, 30]. According to this definition, 165 patients (32%) were at high thromboembolic risk, and 114 patients (22%) were at high haemorrhagic risk. Table 2 shows the distribution of PPS and IBS according to the diagnosis.

**Table 2:** PPS and IBS according to diseases

	Padua Prediction Score (PPS)		Improve Bleeding Score (IBS)	
	< 4 *N* = 349	≥ 4 *N* = 165	< 7 *N* = 400	≥ 7 *N* = 114
AML, n. (%)	109 (31)	49 (31)	104 (26)	54 (47)
ALL, n. (%)	36 (10)	12 (7)	32 (8)	16 (14)
APL, n. (%)	13 (4)	7 (4)	8 (2)	12 (11)
Lymphoma, n. (%)	108 (31)	65 (39)	157 (39)	16 (14)
Multiple Myeloma, n. (%)	47 (14)	20 (12)	62 (16)	5 (4)
Other:				
− malignant, n. (%)	12 (3)	12 (7)	14 (3)	10 (9)
− non-malignant, n. (%)	24 (7)	0	23 (6)	1 (1)

AML; Acute Myeloid Leukemia; ALL: Acute Lymphoblastic Leukemia; APL: Acute Promyelocytic Leukemia

A total of 152 patients (29.6% of the total), including 87 (57.2%) at high thromboembolic risk and 65 (42.8%) at low thromboembolic risk, received pharmacological prophylaxis, whereas 362 patients (70.4% of the total) did not (Table S1).

**Fig. 1:**
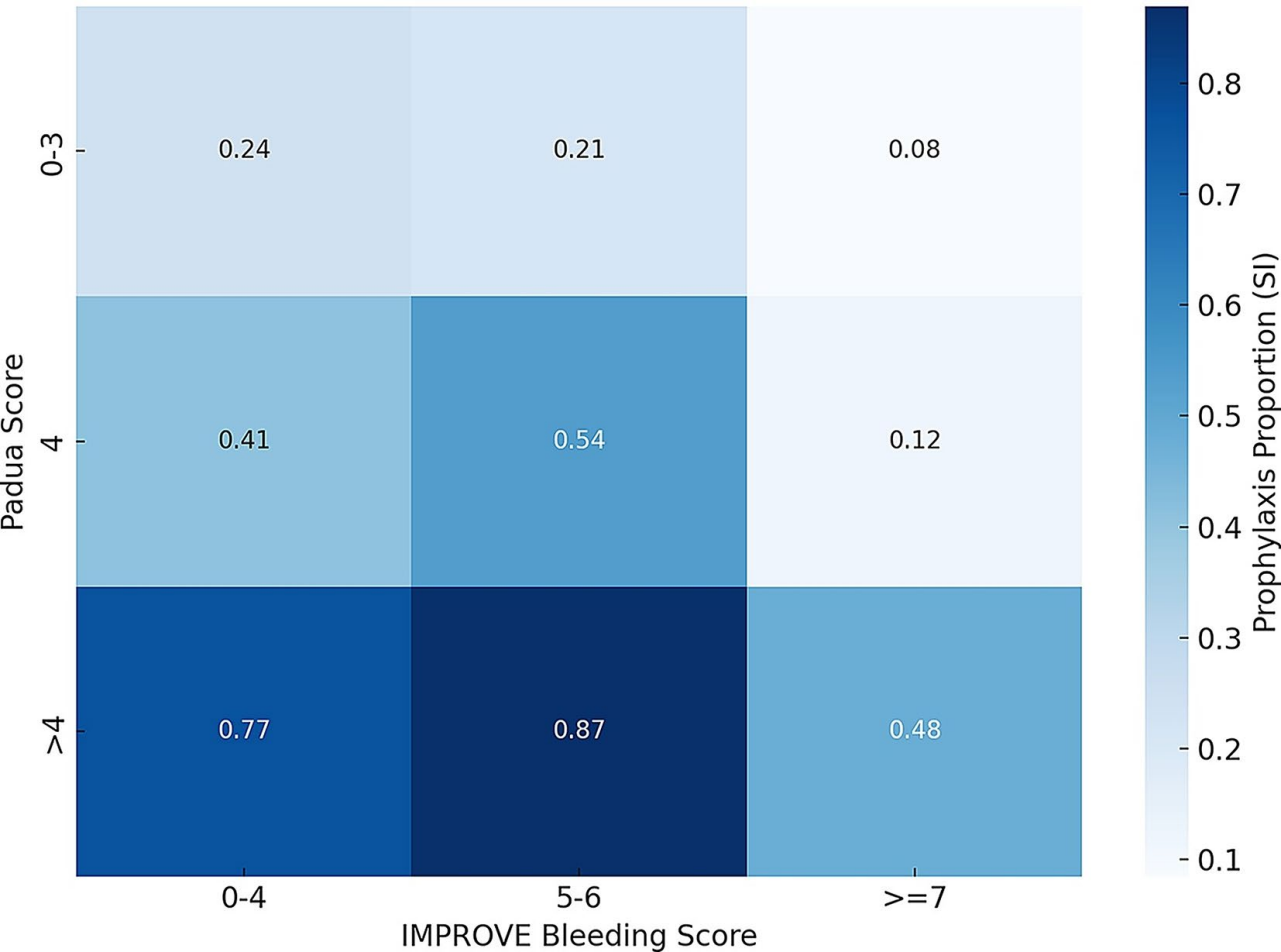
Heatmap of the probability of prophylaxis use according to the Padua prediction score and improve bleeding score values

Figure 1 shows the heatmap of the probability of prophylaxis use according to the PPS and IBS values. Assuming that prophylaxis should have been administered to patients at high thromboembolic and low bleeding risks, respectively, and avoided in other cases, 395 patients (77% of the total) were managed appropriately, while 119 patients (23% of the total) were not. Among the patients in whom prophylaxis was not managed according to the criteria outlined above, 65 out of 119 (54.6%) received prophylaxis despite being at low thromboembolic risk, and 40 out of 119 (33.6%) were not treated despite having high thromboembolic and low bleeding risks, respectively.

Among 52 patients with both high thromboembolic and high bleeding risk, 14 (27%) received prophylaxis, whereas 38 (73%) did not.

Sixteen VTE (6 pulmonary embolisms, 4 catheter related venous thrombosis, 3 distal deep vein thrombosis, 1 renal vein thrombosis, 1 mesenteric vein thrombosis, and 1 cerebral cortical vein thrombosis) were recorded.

Median time to VTE was 18.5 days (IQR 10.5–32.5); 6 VTE occurred during chemotherapy, and the remainders after its completion.

Notably, the 226 patients diagnosed with AL experienced 14 of the 16 reported VTE, with an estimated incidence of 6.2% in this specific subgroup.

No arterial thrombotic events occurred during the study period.

Nine HE (4 gastrointestinal (GI) bleedings, 3 soft tissue hematomas, 1 subdural hematoma, and 1 subarachnoid haemorrhage) were found.

Table 3 represents the distribution of VTE and HE according to the PPS and IBS values. Of note, 11 of the patients experiencing a VTE were at low risk according to the PPS.

**Table 3:** TE e HE (%) on susceptible populations according to PPS/IBS and prophylaxis

	No Prophylaxis		Prophylaxis	
TE	PPS < 4	PPS ≥ 4	PPS < 4	PPS ≥ 4
IBS < 7	6/225 (3)	2/40 (5)	0/62	1/73 (1)
IBS ≥ 7	5/59 (8)	2/38 (5)	0/3	0/14
Total	11/284 (4)	4/78 (5)	0/65	1/87 (1)
*HE*	IBS < 7	IBS ≥ 7	IBS < 7	IBS ≥ 7
PPS < 4	2/225 (1)	4/59 (7)	0/62	0/3
PPS ≥ 4	1/40 (3)	1/38 (3)	0/73	1/14 (7)
Total	3/265 (1)	5/97 (5)	0/135	1/17 (6)

Moreover, most VTE (15 out of 16) occurred in patients who were not receiving pharmacological prophylaxis, 14 of whom with a diagnosis of acute leukaemia.

Prophylaxis was found to be safe, as 8 out of the 9 total HE events occurred in the group that did not receive prophylaxis. The incidence of HE was similar in high-bleeding risk patients receiving prophylaxis compared to those who did not (6% vs. 5%, respectively). The only HE in the prophylaxis group occurred in a patient who was at high risk for both bleeding and thrombosis.

No further statistical analysis was performed on the risk factors for HE, including IBS, due to the low number of events.

**Fig. 2:**
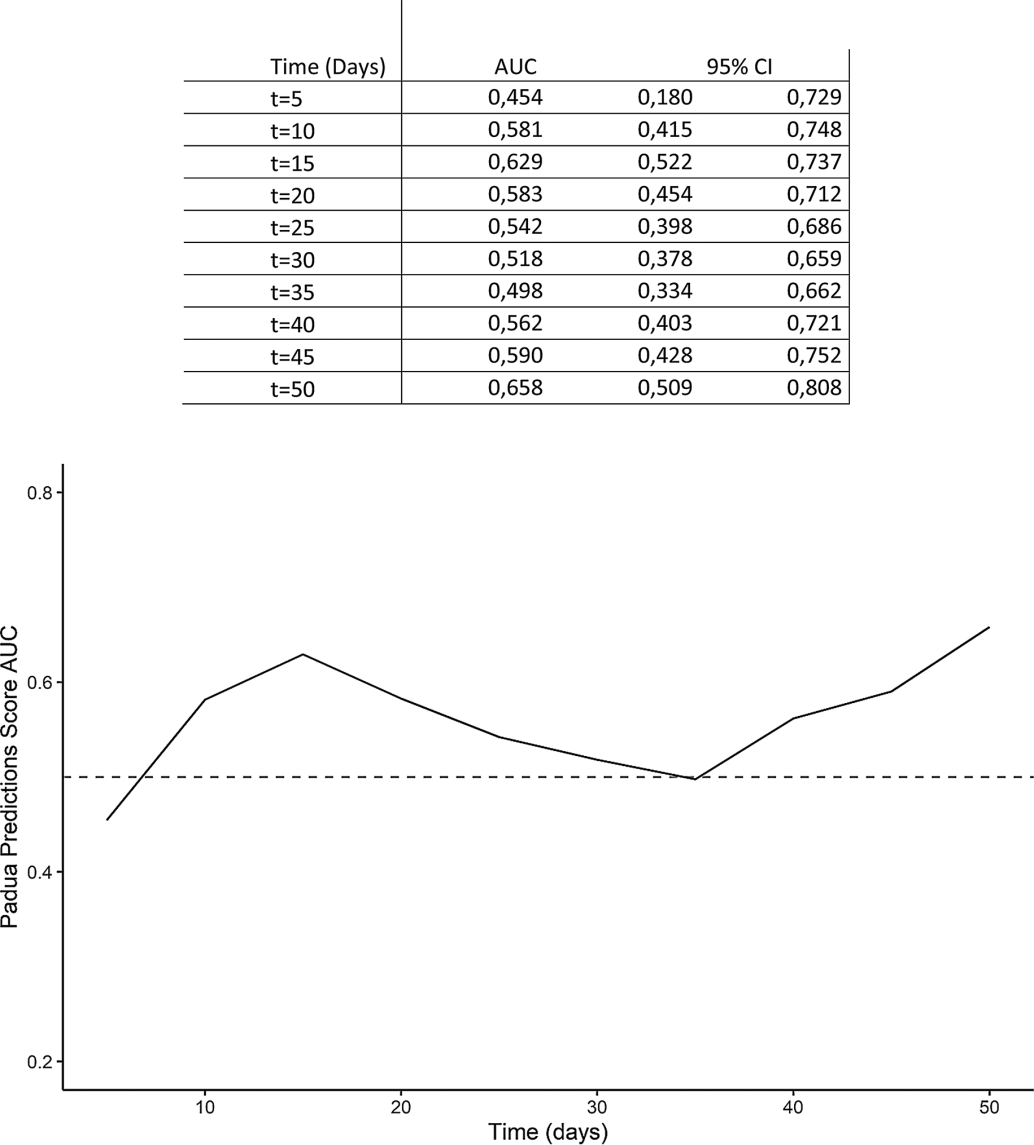
Time-depending Receiver Operating Characteristic (ROC) curves to assess the accuracy of the padua prediction score in predicting in-hospital venous thromboembolism in hematological patients

We computed receiver operating characteristic (ROC) curves to assess the accuracy of the PPS in predicting in-hospital VTE. The area under the curve (AUC), assessed at different times during hospitalization, was poor, ranging from 0.454 (95% Confidence Interval [CI] 0.180 to 0.729) at 5 days to 0.658 (95% CI 0.509 to 0.808) at 50 days (Fig. 2).

Table 4 shows the results of the univariable and multivariable risk regression for VTE in the overall population. In the univariate analysis, the diagnosis of acute leukaemia, particularly acute promyelocytic leukaemia (APL) and acute lymphoblastic leukaemia (ALL), was significantly associated with an increased risk of VTE (HR = 3.66, 95% CI 1.04–12.92, *p* = 0.044 and HR = 3.22, 95% CI 1.11–9.29, *p* = 0.031, respectively), whereas the use of pharmacological prophylaxis significantly reduced the risk of experiencing a VTE (HR = 0.13, 95% CI 0.02–0.97, *p* = 0.047).

**Table 4:** Univariable and multivariable analyses for VTE

Covariates	Univariable		Multivariable	
	HR	*P*	HR	*p*
Sex M/F	0.41 [0.14–1.17]	0.095		
Age	1.00 [0.97–1.04]	0.795		
PLT at admission	1.00 [1.00–1.00]	0.117		
Anticoagulant prophylaxis	*0.13 [0.02–0.97]*	*0.047*	0.22 [0.02–1.93]	0.170
AL	*5.02 [1.12–22.55]*	*0.035*	2.76 [0.55–13.88]	0.218
AML (excluding APL)	0.84 [0.30–2.36]	0.838		
APL	*3.66 [1.04–12.92]*	*0.044*		
ALL	*3.22 [1.11–9.29]*	*0.031*		
Lymphoma	0.18 [0.02–1.35]	0.095		
Multiple Myeloma	0.04 [< 0.01->99.99]	0.434		
Other malignant	1.77 [0.30-13.67]	0.585		
Other non-malignant	0.05 [< 0.01->99.99]	0.764		
Heart and/or respiratory failure	21.17 [< 0.01->99.99]	0.635		
Acute myocardial infarction	20.52 [< 0.01->99.99]	0.760		
Ischemic stroke	20.15 [< 0.01->99.99]	0.967		
Acute infection	1.14 [0.32–4.02]	0.842		
Obesity	2.26 [0.64–8.03]	0.207		
Hormonal treatment	20.54 [< 0.01->99.99]	0.758		
Recent trauma/major surgery < 1 month	2.35 [0.30–18.40]	0.415		
Previous VTE	20.86 [< 0.01->99.99]	0.692		
Reduced mobility (bedrest > 2 days)	0.45 [0.06–3.50]	0.448		
Thrombophilia (congenital or acquired)	22.44 [< 0.01->99.99]	0.600		

PLT: platelet count; AML; Acute Myeloid Leukemia; ALL: Acute Lymphoblastic Leukemia; APL: Acute Promyelocytic Leukemia; VTE= Venous Thromboembolism

The characteristics found to be significant in the univariable analysis, namely anticoagulant prophylaxis and the diagnosis of acute leukaemia, were considered for the multivariable analysis. As shown in Table 4, statistical significance was lost for both variables (HR = 0.22, 95% CI 0.02–1.93, *p* = 0.170 and HR = 2.76, 95% CI 0.55–13.88, *p* = 0.218, respectively).

## Discussion

Patients hospitalized for HD are underrepresented in studies on venous thromboembolism (VTE) prophylaxis, as they carry both high thromboembolic and hemorrhagic risks.Consequently, only limited data are available on the incidence VTE and HE during hospitalization, as well as on the performance of RAM, such as the PPS and the IBS, and on the safety of pharmacological VTE prophylaxis in this population.

We found a non-negligible incidence of venous thromboembolic (3.11%) and hemorrhagic (1.75%) events in our cohort of HD patients, 91% of whom had a diagnosis of HM, at their first hospital admission.

Our finding contrasts with a recently published paper by Carini and colleagues, which reported a VTE incidence of 1.9% in patients with HM admitted to non-ICU wards, and 3.7% in those admitted to the ICU [34]. This discrepancy may be due to a different distribution of hematological diseases in the studies, with a lower rate of patients with AL in their study compared to ours (8.3% vs. 44%).

Of note, 14 of the 16 VTEs occurred in patients with AL, with an estimated incidence of 6.2%.

Table 5 provides a summary of VTE incidence in patients with AL as reported in other studies. Except for the study by Ku, all others reported VTE incidences significantly higher than ours [5, 27–30]. However, it should be noted that the median time to onset of VTE in those studies was longer than in ours, which focused on very early thrombotic events occurring during the first hospitalization, with a median time to thrombotic event of 18.5 days. As a result, the low incidence of thrombotic events in our populations affected by lymphoma and multiple myeloma may appear discordant with previously reported incidence rates. However, we would like to emphasize that our analysis was limited to thromboembolic events occurring during the hospitalization period, in contrast with mostly available data on thrombotic risk in these populations refer to cumulative incidence over longer observation periods, often including outpatient follow-up.

**Table 5:** Previously reported incidence of venous thromboembolism (VTE) in acute leukemia patients

Study [ref]	Patients (*n*)	VTE incidence	Median time to VTE
Ku [5]	626	3% at 3 months	-nr
Al-Ani [27]	425 AML (including APL) 74 ALL	9.6% at 3 months	64 days
Martella [28]	210 AML (excluding APL) 12 high-risk MDS	10% at 6 months	84 days
Paterno [29]	300 AML (excluding APL)	11% at 3 months	82% of events within 45 days from AL diagnosis
Mitrovic [29]	626 AML pts.	11.5% at 6 months	3 months

AML; Acute Myeloid Leukemia; ALL: Acute Lymphoblastic Leukemia; APL: Acute Promyelocytic Leukemia

This initial phase of clinical history in patients with HD, particularly those with AL, remains underrepresented in the current literature.

We believe our findings can help to further clarify the dynamics of thromboembolic risk profiles in these patients.

None of these studies reported the use of pharmacological prophylaxis in the included patients, while acquiring data on this debated topic would be highly valuable, especially in a unique population that simultaneously carries both bleeding and thrombotic risks.

In our study, pharmacological prophylaxis was prescribed to about one-third of our patients and was found to be both effective and safe, as 15 out of the 16 VTE cases occurred in patients who were not receiving prophylaxis, and its use did not increase the risk of HE.

Moreover, we found that only 9% of patients diagnosed with AL received pharmacological prophylaxis, compared to 45% of those with other diagnoses. This difference may account for the different incidence of VTE between the two groups (6.2% vs. 3.1%, respectively).

In about three-quarters of cases, pharmacological prophylaxis was appropriately prescribed based on thromboembolic and hemorrhagic risks estimated by the PPS and IBS. However, 11 VTE cases were recorded in patients classified as low risk for thromboembolism according to the PPS, suggesting that this prediction score may perform inadequately in HD patients. This observation was objectively confirmed by the unsatisfactory AUC values found in our study, indicating that the PPS may not be suitable for estimating VTE risk in hospitalized hematology patients.

This finding is not surprising, as the PPS was developed and validated in a general population of patients hospitalized in internal medicine wards, which differs substantially from our study population. Additionally, prior studies have shown the PPS to perform poorly when applied to specific patient groups, such as those with sepsis [35].

Patients hospitalized for HM present a high degree of clinical complexity, with a particularly delicate balance between hemorrhagic and thrombotic risks. Therefore, validated tools to estimate both thrombotic and hemorrhagic risks—and thus guide the use of pharmacological prophylaxis—would be highly beneficial in practice.

We aimed to develop a RAM specifically tailored to hospitalized HD patients by identifying other risk factors significantly associated with the development of VTE. However, in this study, we were unable to identify such factors. While univariable analysis suggested that patients diagnosed with acute promyelocytic leukemia or acute lymphoblastic leukemia might be at a higher risk of thromboembolism, this finding was not confirmed by subsequent multivariable analysis, likely due to the small number of thrombotic events recorded.

Furthermore, as mentioned above, the vast majority of VTE cases occurred in patients diagnosed with AL who were not receiving prophylaxis. The alignment between the outcome and the distribution of these two variables may reduce the ability of the statistical model to differentiate their individual effects. Therefore, the results of the multivariable model should be interpreted with caution.

We acknowledge that our study has certain limitations, including its single-centre and retrospective design, the low number of recorded events and the lack of some important information regarding concurrent risk factors for VTE, such as chemotherapy administered or phases of therapy of hemopathy.

However, several aspects of this study represent strengths. Primarily, we enrolled consecutive patients hospitalized at the same center, with shared protocols and consistent awareness of thromboembolic risk among all healthcare providers. Another noteworthy aspect is the study’s focus on the first hospitalization, which provides a clear picture of thromboembolic risk at the onset of these patients’ clinical course.

Large, prospective multicentre studies are needed to gather more robust data on the critical issue of VTE risk and the use of pharmacological prophylaxis in patients hospitalized for HD.

## Electronic supplementary material

Below is the link to the electronic supplementary material.

Supplementary Material 1

## Data Availability

Data collected and analyzed for the current study are available upon reasonable request and approval of the study investigators and of the Ethics Committee.

## References

[1] Ay C, Pabinger I, Cohen AT (2017) Cancer-associated venous thromboembolism: burden, mechanisms, and management. Thromb Haemost 117(2):219–230. 10.1160/TH16-08-0615 27882374 10.1160/TH16-08-0615

[2] Mulder FI, Horváth-Puhó E, van Es N et al (2021) Venous thromboembolism in cancer patients: a population-based cohort study. Blood 137(14):1959–1969. 10.1182/blood.2020007338 33171494 10.1182/blood.2020007338

[3] Falanga A, Marchetti M (2023) Cancer-associated thrombosis: enhanced awareness and pathophysiologic complexity. J Thromb Haemost 21(6):1397–1408. 10.1016/j.jtha.2023.02.029 36931602 10.1016/j.jtha.2023.02.029

[4] Khorana AA, Mackman N, Falanga A et al (2022) Cancer-associated venous thromboembolism. Nat Rev Dis Primers 8(1):11. 10.1038/s41572-022-00336-y 35177631 10.1038/s41572-022-00336-y

[5] Ku GH, White RH, Chew HK, Harvey DJ, Zhou H, Wun (2009) Venous thromboembolism in patients with acute leukemia: incidence, risk factors, and effect on survival. Blood 113(17):3911–3917. 10.1182/blood-2008-08-175745 10.1182/blood-2008-08-175745 PMC2673120 19088376

[6] Falanga A, Marchetti M, Russo L (2012) Venous thromboembolism in the hematologic malignancies. Curr Opin Oncol 24(6):702–710. 10.1097/CCO.0b013e3283592331 23014188 10.1097/CCO.0b013e3283592331

[7] Wang TF, Leader A, Sanfilippo K (2022) Review on thrombosis and bleeding in hematological malignancy. Best Pract Res Clin Haematol 35(1):101353. 10.1016/j.beha.2022.101353 36030068 10.1016/j.beha.2022.101353

[8] Casini A, Fontana P, Lecompte T (2013) Thrombotic complications of myeloproliferative neoplasms: risk assessment and risk guided management. J Thromb Haemost 11:1215–1227. 10.1111/jth.12265 23601811 10.1111/jth.12265

[9] Kristinsson SY, Pfeiffer RM, Björkholm M, Schulman S, Landgren O (2012) Thrombosis is associated with inferior survival in multiple myeloma. Haematologica 97(10):1603–1607. 10.3324/haematol.2012.064444 22511493 10.3324/haematol.2012.064444 PMC3487563

[10] Mahajan A, Wun T, Chew H, White RH (2014) Lymphoma and venous thromboembolism: influence on mortality. Thromb Res 133(Suppl 2):S23. 10.1016/S0049-3848(14)50004-7 24862141 10.1016/S0049-3848(14)50004-7

[11] Poh C, Brunson A, Keegan T, Wun T, Mahajan A (2020) Incidence of Upper Extremity Deep Vein Thrombosis in Acute Leukemia and Effect on Mortality. TH Open 2020;4(4):e309-e317. 10.1055/s-0040-1718883 10.1055/s-0040-1718883 PMC7593117 33134806

[12] Schoen MW, Carson KR, Luo S et al (2020) Venous thromboembolism in multiple myeloma is associated with increased mortality. Res Pract Thromb Haemost 4(7):1203–1210. 10.1002/rth2.12411 33134785 10.1002/rth2.12411 PMC7590313

[13] Goncalves I, Lewis C, Grainger B, Dring R, Lee N, Pasricha SR, Szer J, Mason K (2024) Thrombosis in patients with immune thrombocytopenia: incidence, risk, and clinical outcomes. Res Pract Thromb Haemost 8(1):102342. 10.1016/j.rpth.2024.102342 38444612 10.1016/j.rpth.2024.102342 PMC10912689

[14] Tse B, Lim G, Sholzberg M, Pavenski K (2020) Describing the point prevalence and characteristics of venous thromboembolism in patients with thrombotic thrombocytopenic purpura. J Thromb Haemost 18(11):2870–2877. 10.1111/jth.15027 33448602 10.1111/jth.15027

[15] Khorana AA, Kuderer NM, Culakova E, Lyman GH, Francis CW (2208) Development and validation of a predictive model for chemotherapy-associated thrombosis. Blood 111(10):4902–4907. 10.1111/jth.15027 10.1182/blood-2007-10-116327 PMC2384124 18216292

[16] Ay C, Dunkler D, Marosi C et al (2010) Prediction of venous thromboembolism in cancer patients. Blood 116(24):5377–5382. 10.1182/blood-2010-02-270116 20829374 10.1182/blood-2010-02-270116

[17] Verso M, Agnelli G, Barni S, Gasparini G, LaBianca R (2012) A modified Khorana risk assessment score for venous thromboembolism in cancer patients receiving chemotherapy: the protecht score. Intern Emerg Med 7:291–292. 10.1007/s11739-012-0784-y 22547369 10.1007/s11739-012-0784-y

[18] Gerotziafas GT, Taher A, Abdel-Razeq H, COMPASS–CATWorking Group et al (2017) A predictive score for thrombosis associated with breast, colorectal, lung, or ovarian cancer: the prospective COMPASS Cancer-Associated thrombosis study. Oncologist 22(10):1222–1231. 10.1634/theoncologist.2016-0414 28550032 10.1634/theoncologist.2016-0414 PMC5634762

[19] Pabinger I, van Es N, Heinze G et al (2018) A clinical prediction model for cancer-associated venous thromboembolism: a development and validation study in two independent prospective cohorts. Lancet Haematol 5(7):e289–e298. 10.1016/S2352-3026(18)30063-2 29885940 10.1016/S2352-3026(18)30063-2 PMC7338218

[20] Muñoz A, Ay C, Grilz E et al (2023) A Clinical-Genetic risk score for predicting Cancer-Associated venous thromboembolism: A development and validation study involving two independent prospective cohorts. J Clin Oncol 41(16):2911–2925. 10.1200/JCO.22.0025 36730884 10.1200/JCO.22.00255 PMC10414737

[21] Cella CA, Knoedler M, Hall M et al (2023) Validation of the ONKOTEV risk prediction model for venous thromboembolism in outpatients with Cancer. JAMA Netw Open 6(2):e230010. 10.1001/jamanetworkopen.2023.0010 36795409 10.1001/jamanetworkopen.2023.0010 PMC9936336

[22] López SA, Tejeda Ramón MC, Morales Helguera A et al (2023) Validation of venous thromboembolism predictive model in hematologic malignancies. Ann Hematol 102(12):3613–3620. 10.1007/s00277-023-05463-4 37782372 10.1007/s00277-023-05463-4

[23] Antic D, Milic N, Nikolovski S, Todorovic M, Bila J, Djurdjevic P et al (2016) Development and validation of multivariable predictive model for thromboembolic events in lymphoma patients. Am J Hematol 91(10):1014–1019. 10.1002/ajh.24466A 27380861 10.1002/ajh.24466

[24] Sanfilippo KM, Luo S, Wang TF et al (2019) Predicting venous thromboembolism in multiple myeloma: development and validation of the IMPEDE VTE score. Am J Hematol 94(11):1176–1184. 10.1002/ajh.25603 31379000 10.1002/ajh.25603 PMC7058359

[25] Chen Y, Lei H, Wang W, Zhu J et al (2022) Characteristics and predictors of venous thromboembolism among lymphoma patients undergoing chemotherapy: A cohort study in China. Front Pharmacol 13:901887. 10.3389/fphar.2022.901887 35677441 10.3389/fphar.2022.901887 PMC9168459

[26] Hohaus S, Bartolomei F, Cuccaro A, Maiolo E, Alma E, D’Alò F et al (2020) Venous thromboembolism in lymphoma: risk stratification and antithrombotic prophylaxis. Cancers (Basel) 12(5):1291. 10.3390/cancers12051291 32443753 10.3390/cancers12051291 PMC7281118

[27] Al-Ani F, Wang YP, Lazo-Langner A (2020) Development of a clinical prediction rule for venous thromboembolism in patients with acute leukemia. Thromb Haemost 120(2):322–328. 10.1055/s-0039-3400303 31893562 10.1055/s-0039-3400303

[28] Martella F, Cerrano M, Di Cuonzo D, Secreto C, Olivi M, Apolito V et al (2022) Frequency and risk factors for thrombosis in acute myeloid leukemia and high-risk myelodysplastic syndromes treated with intensive chemotherapy: a two centers observational study. Ann Hematol 101(4):855–867. 10.1007/s00277-022-04770-6 35128571 10.1007/s00277-022-04770-6

[29] Paterno G, Palmieri R, Forte V et al (2022) Predictors of early thrombotic events in adult patients with acute myeloid leukemia: A Real-World experience. Cancers (Basel) 14(22):5640. 10.3390/cancers14225640 36428732 10.3390/cancers14225640 PMC9688263

[30] Mitrovic M, Pantic N, Bukumiric Z et al (2024) Venous thromboembolism in patients with acute myeloid leukemia: development of a predictive model. Thromb J 22(1):37. 10.1186/s12959-024-00607-6 38632595 10.1186/s12959-024-00607-6 PMC11022429

[31] Horner D, Goodacre S, Davis S, Burton N, Hunt BJ (2013) Which is the best model to assess risk for venous thromboembolism in hospitalised patients? BMJ 373:n1106. 10.1136/bmj.n1106 10.1136/bmj.n1106 34045235

[32] Barbar S, Noventa F, Rossetto V et al (2010) A risk assessment model for the identification of hospitalized medical patients at risk for venous thromboembolism: the Padua prediction score. J Thromb Haemost 8:2450–2457. 10.1111/j.1538-7836.201004044.x 20738765 10.1111/j.1538-7836.2010.04044.x

[33] Decousus H, Tapson VF, Bergmann JF, IMPROVE Investigators et al (2011) Factors at admission associated with bleeding risk in medical patients: findings from the IMPROVE investigators. Chest 139(1):69–79. 10.1378/chest.09-3081 20453069 10.1378/chest.09-3081

[34] Carini FC, Angriman F, Scales DC, Munshi L, Burry LD, Sibai H, Mehta S, Ferreyro BL, SELECTION study group (2024) Venous thromboembolism in critically ill adult patients with hematologic malignancy: a population-based cohort study. Intensive Care Med 50(2):222–233. 10.1007/s00134-023-07287-2 38170226 10.1007/s00134-023-07287-2

[35] Vardi M, Ghanem-Zoubi NO, Zidan R, Yurin V, Bitterman H (2013) Venous thromboembolism and the utility of the Padua prediction score in patients with sepsis admitted to internal medicine departments. J Thromb Haemost 11:467–473. 10.1111/jth.12108 23279085 10.1111/jth.12108

