# Supplementary material for: Venous thromboembolic risk in hematological hospitalized patients: a retrospective study
Source: Ann Hematol. 2025 May 6;104(5):2963–71. doi: 10.1007/s00277-025-06397-9 (PMC12141132; doi:10.1007/s00277-025-06397-9)
Supplement: Supplementary file 1 — Supplementary Material 1 [file 277_2025_6397_MOESM1_ESM.docx]

**SUPPLEMENTARY MATERIAL**

**Table S1**

*Distribution of patients receiving or not prophylaxis according to PPS/IBS.*

|  | No Prophylaxis | | Prophylaxis | |
| --- | --- | --- | --- | --- |
|  | PPS < 4  n (%) | PPS ≥ 4  n (%) | PPS < 4  n (%) | PPS ≥ 4  n (%) |
| IBS < 7 | 225 (62) | 40 (11) | 62 (41) | 73 (48) |
| IBS ≥ 7 | 59 (16) | 38 (11) | 3 (2) | 14 (9) |

*PPS: Padua Prediction Score; IBS: Improve Bleeding Score*
